# Supplementary material for: The AlkB Homolog SlALKBH10B Negatively Affects Drought and Salt Tolerance in Solanum lycopersicum
Source: Int J Mol Sci. 2023 Dec 22;25(1):173. doi: 10.3390/ijms25010173 (PMC10778744; doi:10.3390/ijms25010173)
Supplement: Supplementary file 1 [file ijms-25-00173-s001.zip › Supplementary Figure.pdf]

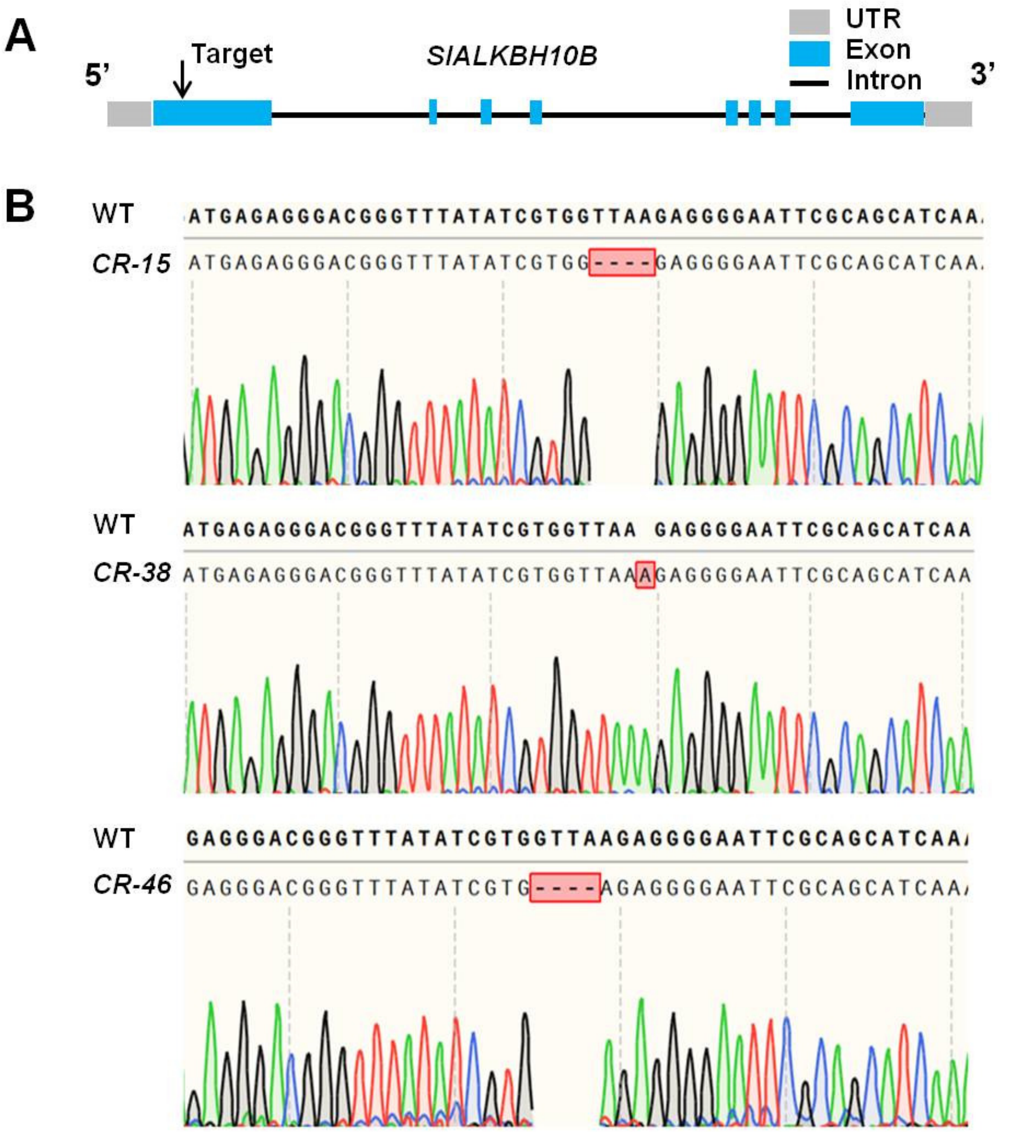


**Figure S1. Knockout site and sequence alignment. (A)** The gene knockout site was located in the first exon of *SlALKBH10B* and near the ATG start codon. **(B)** Genomic sequenes flanking the knockout site of wild-type and the mutant lines.
